# Stress levels and coping strategy of nursing students in online learning during COVID-19 Pandemic. A mixed-methods study*

**DOI:** 10.17533/udea.iee.v41n2e09.

**Published:** 2023-08-24

**Authors:** Evelyn Hemme Tambunan, Rosnancy Renolita Sinaga

**Affiliations:** 1 . Ph.D. NED Assistant Professor. Faculty of Nursing Science, Universitas Advent Indonesia, Indonesia. Email: evelyntambunan@unai.edu. https://orcid.org/0000-0002-9856-4001 Faculty of Nursing Science Universitas Advent Indonesia Indonesia evelyntambunan@unai.edu; 2 . MSN. Assistant Professor. School of Nursing Surya Nusantara, Pematangsiantar, Indonesia. Email: rosnancy.sinaga@suryanusantara.ac.id https://orcid.org/0000-0002-9539-3585 School of Nursing Surya Nusantara Pematangsiantar Indonesia rosnancy.sinaga@suryanusantara.ac.id https://orcid.org/0000-0002-9539-3585

**Keywords:** COVID-19, students, nursing, anxiety, depression, adaptation, psychological, internet-based intervention, COVID-19, estudiantes de enfermería, ansiedad, depresión, adaptación psicológica, intervención basada en la internet, COVID-19, estudantes de enfermagem, ansiedade, depressão, adaptação psicológica, intervenção baseada em internet

## Abstract

**Objective:**

To explain the stress level and coping strategies of nursing students in online learning during Covid-19 pandemic.

**Methods:**

Explanatory sequential mixed method QUAN-QUAL study conducted at a private university in Bandung, Indonesia. Of the 260 nursing students, 157 consented to participate and answered a Depression Anxiety Scale-42 (DDAS-42) and The Ways of Coping in the Indonesian version. The participants of the quantitative phase with the indicative of stress were interviewed individually (*n*=17) to provide an in-depth understanding of the students’ experiences of stress and coping strategy in online learning.

**Results:**

Almost one out of two students (47.1%) had some level of stress (16% between severe and extremely severe). Most nursing students (45.9%) used emotion focused coping strategies. Stress level was significantly higher among female students and internship academic level (*p*<0.05). Nursing students’ sources of stress were new experiences and hindrances to online learning. Coping strategies included seeking support and positive acceptance.

**Conclusion:**

A high proportion of nursing students experienced stress during their education process in COVID-19 times; they used specially emotion-focused coping strategies to reduce barriers to online learning.

## Resumen

**Objetivo:**

Describir el nivel de estrés y las estrategias de afrontamiento de los estudiantes de enfermería en el aprendizaje en línea durante la pandemia de Covid-19.

**Métodos:**

Estudio secuencial de método mixto QUAN-QUAL realizado en una universidad privada de Bandung, Indonesia. De los 260 estudiantes de enfermería, 157 consintieron en participar y respondieron las escalas de Depresión y Ansiedad-42 (DDAS-42) y la de Formas de Afrontamiento. Se entrevistó individualmente a los participantes que en la fase cuantitativa mostraron indicios de estrés (*n*=17) para conocer en profundidad sus experiencias y las estrategias de afrontamiento empleadas.

**Resultados:**

Casi uno de cada dos estudiantes (47.1%) presentó algún nivel de estrés (16% entre severo y extremadamente severo). El 45.9% de los estudiantes utilizo estrategias de afrontamiento centradas en las emociones. El nivel de estrés fue significativamente mayor en el sexo femenino y en el nivel académico de internado (*p*<0.05). Las fuentes fueron las nuevas experiencias y los obstáculos para el aprendizaje en línea. Las estrategias de afrontamiento incluyeron la búsqueda de apoyo y la aceptación positiva.

**Conclusión:**

Una alta proporción de estudiantes de enfermería experimentó estrés durante su proceso de formación en tiempos de COVID-19, ante lo cual utilizó estrategias de afrontamiento centradas en las emociones para reducir las barreras al aprendizaje en línea.

## Resumo

**Objetivo:**

Descrever o nível de estresse e as estratégias de enfrentamento de estudantes de enfermagem no ensino online durante a pandemia do Covid-19.

**Métodos:**

Estudo sequencial de método misto QUAN-QUAL realizado em uma universidade particular em Bandung, Indonésia. Dos 260 estudantes de enfermagem, 157 consentiram em participar e responderam às escalas de Depressão e Ansiedade-42 (DDAS-42) e Formas de Enfrentamento. Os participantes que na fase quantitativa apresentaram sinais de estresse foram entrevistados individualmente (*n*=17) para conhecer a fundo as experiências de estresse e as estratégias de enfrentamento utilizadas.

**Resultados:**

Quase um em cada dois alunos (47.1%) apresentou algum nível de estresse (16% entre grave e extremamente grave). 45.9% dos alunos usaram estratégias de enfrentamento focadas na emoção. O nível de estresse foi significativamente maior no sexo feminino e no nível acadêmico do internato (*p*<0.05). As fontes de estresse dos estudantes de enfermagem foram novas experiências e obstáculos para o aprendizado online. As estratégias de enfrentamento incluíram a busca de apoio e aceitação positiva.

**Conclusão:**

Uma alta proporção de estudantes de enfermagem experimentou estresse durante seu processo de formação em tempos de COVID-19, para os quais eles usaram especialmente estratégias de enfrentamento focadas na emoção para reduzir as barreiras ao aprendizado online.

## Introduction

Online learning is defined as learning experiences in synchronous or asynchronous contexts employing a variety of devices with internet connection, such as cell phones, computers, and other devices_._^1^ Students can learn and engage with professors and other students from anywhere. While synchronous environment of learning needs a live and real time interactions between educators and learners, an asynchronous is more in another learning system and forums that promote learners independence learning management.^2^^,^^3^ Likewise in nursing education, the importance of technology is well understood. Teaching has successfully incorporated technology into the learning process in order to prepare nursing students to function in a technology-driven health setting. Blended learning, distance learning, e-learning are an evolution in nursing education that incorporate technology in learning.^4^^-^^6^

The Coronavirus 2019 (Covid-19) pandemic globally had various impacts on the higher education climate. Social distancing initiatives were undertaken by education sectors. Online learning system was implemented in most higher education institutions.^7^ Data presented by UNESCO showed that around 1.5 billion students enrolled worldwide are forced to at-home distance learning. Several countries initiated to undertake this method include Indonesia.^8^ Since the first two positive cases reported in Indonesia on March second 2020, the fatality rate has continuously increased. Thus, the president of Indonesia Joko Widodo suggested “red zone” epicenters and national lock down. The educational system was corroborated with Indonesia Ministry of Education advisory No 262/E.E2/KM/2020 released on March 23, 2020. The Ministry of Education advisory suggested the online learning alternatives during COVID-19 pandemic must ensure the learning activities.^9^

Undoubtedly, this period of Covid-19 pandemic has been stressful for nursing students. Significant levels of stress among higher education students have been reported worldwide, given that during the years of Covid-19 pandemic, particularly stressful (44.4% ) and also very stressful (47.2%).^10^ Specifically, online learning is a learning system that is not commonly implemented in Indonesia. Nursing students may experience a great deal of stress as a result of abruptly switching from traditional to online learning.^11^ Stress occurs when a person thinks that the demands are greater than his or her personal and social resources. As a result, whether or not a situation is frightening is determined by the individual's perception of it. Nursing students experience challenges as classes go online, such as being unable to concentrate and having difficulty participating, writing projects, taking tests, and achieving academic deadlines.^12^ In addition, that challenges faced by students include lack of in-person interaction, distractions and time management, lack of a systematic schedule, stress and psychological pressure, missing the traditional university environment and lack of access to external learning resources.^13^

Coping strategy refers to an individual’s responses to stressors. Coping strategies are stabilizing techniques for assisting individuals in maintaining psychological adaptation during stressful situations.^14^ In addition coping techniques defined as either problem-based or emotion-based. In the face of a stressful event, both coping mechanisms are applied, although their effectiveness varies. Problem-focused coping seeks to alleviate distress by actively managing a stressor. Obtaining information about the stressful circumstance and its potential implications is required for this method.^15^ People that employ this method attempt to prioritize their activities based on their importance and to manage their activities on a timely basis.^16^ On the other hand, emotion-focused coping is aimed at dealing with the stressor's emotions and feelings. Finding techniques to manage emotions and being hopeful when confronted with stressful events are two of these strategies. People who use this approach to manage their emotions may express emotions such as rage or disappointment.^17^

Previous studies have indicated that while engaging in online learning, nursing students often used coping strategies in different ways. Nursing students have demonstrated remarkable resilience as one of their coping methods throughout Covid-19 outbreak. The learners have used humor, which studies associate with lower to moderate anxiety levels.^18^ Additionally, diverse coping strategies adopted by nursing students included being married, use of emotional social support, acceptance positive reinterpretation and growth and behavioral disengagement.^19^ Prior studies have explored the experience of nursing students in online learning using a qualitative research design and the stress level and coping strategies of nursing students in response to the online learning using cross-sectional design.^20^ Therefore, this study used a mixed method design to explore the stress levels and coping strategy of nursing students in online learning during Covid-19 outbreak in a private nursing school.

## Methods

Design. This study used an explanatory sequential mixed-method design. Separate quantitative and qualitative data were assessed, but they were combined and discussed. While the quantitative phase was based on surveys, the qualitative phase was based on in-depth interviews using semi-structure questions to explore the various stress sources and coping strategies adopted in the online learning environment. 

Setting and participants. Nursing students were recruited from one nursing school of a private university in Bandung, Indonesia, which was expeditiously running online learning due to Covid-19 outspread provision from March 2020. The inclusion criteria were students in bachelor nursing program both in academic and internship stage who experienced online learning in full semesters of 2020/2021 academic year, understood the study purpose, agreed to participate in this study and signed the consent form. A total of 260 nursing students from the bachelor nursing program, and 157 nursing students completed the questionnaire, while 17 bachelor students participated in face-to-face interviews. 

Data Collection. Questionnaires of quantitative measurement in this study included basic characteristics, the Depression Anxiety Stress Scale-42 (DASS-42) and The Ways of Coping. DASS-42 was adopted from Psychology Foundation of Australia and The Ways of Coping was adopted from Folkman and Lazarus. Both questionnaires were translated to the Indonesian version and the scales have been shown to have good reliability in many populations. While the reliability of Chronbach’s alpha stress dimension of DASS-42 was 0.89, The Ways of Coping was 0.81. Score indicator for DASS-42 was normal (0-14), mild (15-18), moderate (19-25), severe 26-33) and extremely severe (>34). While coping indicators are described by coping focused on problems, emotions and used together in a balanced way.^21^^,^^22^ Using semi-structured questions, a qualitative data guide was created. The participants were encouraged to share freely about their experience of stress and coping strategy used during the period of online learning amid Covid-19 pandemic. The interview questions were: (1) What are the main stressors in online learning during the Covid-19 pandemic? (2) How do you manage stress while engaging in online learning during Covid-19 pandemic? (4) Could you describe how you expect the stress to be reduced?

Data Analysis. Variables were analyzed using descriptive statistics which included: percentage, frequencies, mean and standard deviation (SD) for gender, age stress levels and coping strategies utilization. Nursing student’s *t*-test were used to test the differences in means of stress and coping strategies scores by gender and academic levels. The significant level was set at *p*<0.05. Whilst the qualitative data were analyzed using content analysis according to Creswell.^23^ The qualitative data was read numerous times in order to fully comprehend the responses. Units that met the objectives were identified and grouped together into shorter passages. To summarize the text further, they were coded together and grouped into categories and subcategories. After qualitative data analysis, a theme developed. 

Rigor. Credibility involves establishing the truth of the qualitative research study’s findings. In this study, researchers used a peer debriefed strategy to ensure the trustworthiness of the qualitative data gathered. During and after the data gathering and analysis process, the peer debriefed was asked for some inputs for general methodology, transcripts and final report. Afterwards, researchers provided the feedback to enhance credibility and ensure validity of the investigation. 

Ethical Consideration. This study was conducted in accordance with national and international standards for research involving human subjects, including approval by the Institutional Review Board (IRB) of Faculty of Nursing Science (KEPK No: 158/EKS-SU-VIII/20). All participants signed an informed consent form.

## Results

### The results of the quantitative phase

Univariate analysis shows the description of characteristics of the respondent, distribution of stress level and distribution of coping strategy. The number of respondents was as many as 157 students, the mean age of bachelor nursing student was 21.7 years, and most of the respondents were female (67.5%). Meanwhile, based on the academic year, more respondents were at the internship level (32.5%). Analysis on stress levels distribution, the finding showed almost one out of two students (47.1%) had some level of stress (16% between severe and extremely severe). Based on the score ranges from the stress level (DASS-42), the mean scores of stress for all students were found at moderate level 23.42 (SD=1.78). Analysis on coping strategy distribution, most nursing students (45.9%) used emotion focused coping strategies with the mean score at 20.48 (SD=4.23). Bivariate analyses showed the relationship between gender and academic level among nursing students with their mean of stress and coping strategies are shown in Table 1. Statistically significant relationship was found between mean stress level with gender and academic level, where the mean scores were higher in the female group than that in male group. Likewise in academic level, internship level was found highest in the mean score among academic level groups. The ways of coping were not significantly different by both gender and academic level. 

**Table 1:** Mean scores of Stress Levels and The Ways of Coping scales by gender and academic level

Variables	***n* (%)**	Stress Levels Mean ± SD	The Ways of Coping Mean ± SD
Gender			
Male	51 (32.5)	13.13 (7.43)	19.32 (6.92)
Female	106 (67.5)	24.67 (7.09)	20.56 (7.05)
*p-value*		0.005	0.092
Academic level			
Sophomore	28 (17.8)	13.13 (7.28)	21.90 (6.79)
Junior	33 (21.0)	14.66 (8.28)	19.62 (7.62)
Senior	45 (28.7)	13.40 (7.35)	23.80 (8.75)
Internship	51 (32.5)	26.27 (6.14)	22.83 (6.63)
*p-value*		0.012	0.441

### The results of the qualitative phase

The participants ranged in age from 18-33 years (9 females and 8 males.) The results of the qualitative data were classified into two topics: Stress Sources and Coping Strategies. The themes that emerged from each of them, with their respective categories and subcategories, are detailed below and are also available in Table 2. 

**Table 2:** The results of qualitative data analysis

Theme	Category	Subcategory
Topic: Stress sources		
*New experience*	Ineffective learning process	Virtual media replacing clinical
		practice
		Less interactive learning
		Lack of concentration
	Ineffective interaction	Lack of cooperation
		Ineffective communication
*Hindrances to online learning*	External impediments	Academic workload
		Technology impediment
		Remote area
	Internal factor declined	Internal learning motivation weakened
Topic: coping strategies		
*Seeking support*	Government support	Effective use of quota support
	Faculty support	Trying to get closer with faculty
		Enjoying the learning process
	Peer support	Seeking cooperation and collaboration
		Joke with each other
	Family support	Receive support from family members
*Positive acceptance*	Personal adaptation	Respond positively
		Behave positively

### Experience Theme

This theme contained experiences of bachelor nursing students’ stress while engaging in online learning during COVID-19 pandemic era. In the interviews, nursing students in the internship phase expressed that the learning process during online learning was not effective. The virtual media replacing clinical practice was expressed by nursing students at the internship phase. They revealed that clinical practice should be directly involved in those activities on behalf of clients, yet replaced by online learning. They also worried that they are disadvantaged if their practical skills are inadequate compared to those who practice more in hospitals. Students also feel that online learning is less able to fulfill competencies and demands of clinical learning. 

On the other hand, nursing students in the academic phase described an ineffective learning process such as less interactive learning. This subcategory is exemplified by statements such as: *Like before, we couldn't see the lecturer's expression directly, the lecturer couldn't observe whether we understood it or not, that's what I heard from lecturers like that (Participant 2)*. Students in the academic phase also revealed that online learning tends to make students passive. One student explained: *Anyway, if a lecturer asks, sometimes if you want to answer, you have to switch on the microphone first, so we're kind of lazy, so we're more passive in my opinion (Participant 7)*.

In the subcategory “lack of concentration,” it was expressed that nursing students could not focus on learning. One nursing student at academic phase stated: *This online system is also difficult for us to adapt at the beginning, we couldn't meet friends, and maybe sometimes when the lecturer is teaching, I play on my cell phone and then chat with friends who are here, just like that so I can't focus (Participant 11)*. One internship phase nursing student reflected: F*or me, it's quite reduced, because the concentration is not fully there. Because we can do other things, so we don't focus as usual, which is normal (Participant 16).*

In the theme “new experience,” the other category namely “ineffective interaction” was found. It was due to the lack of cooperation among students as told by one nursing student at the academic stage: *But for some people it's more difficult because I don't know them, meaning we've never met and then being asked to say how it's like all out is a bit difficult, so I think it's like that, the collaboration isn't that good (Participant 1).* While one nursing student at the internship stage expressed: *For students, in my opinion, collaboration is reduced online, we students are complicated to zoom in, spending quota, it’s different from offline, we can get together to work together (Participant 13).*

In the subcategory of students who remarked “ineffective communication,” students experienced barriers in communicating as stated: *Because we don't meet face to face, there is sometimes a misunderstanding between what the lecturer wants and what the students get. If we want to ask, it’s really complicated, we have to raise our hand first” (Participant 4).* Another student reflected: C*ommunication between lecturers and students is a bit interrupted, that's because it might be difficult to read the gestures, for example in offline class, maybe the lecturers can directly see, if in online classes this is more limited (Participant 6)*. In addition, the nursing student and lecturer interaction was constrained. One student at the academic stage stated: *So far, there has been less interaction in online classes, sometimes the lecturers call out like that, so it's like we are quiet and dry (Participant 5).*

### Hindrances to Online Learning Theme

This theme was due to challenges experienced by nursing students in online learning during Covid-19 pandemic era. The “external impediments,” and “internal factor declined,” were found as a category of theme. In the subcategory of “academic workload,” it was shown that nursing students complained about difficulties in handling assignments. One nursing student at an academic stage expressed: *The assignments are so many, then the deadline for collecting assignments is not far or tight, and sometimes doesn’t understand what the lecturer explained (Participant 8*). Another nursing student also reflected: *It was at first so stressful, I was given a lot of assignments, sometimes also a lot of materials (Participant 10).*

The sub category of “technology impediment,” explained how technology has limitations in facilitating online learning. Various technology challenges in the form of limitations of its technological tools and also the limitations of the technology users. One nursing student at an academic stage stated: *Gaining knowledge is difficult. Then sometimes on the internet, whether it's the lecturer or us, the internet has errors, so the lecturer's voice becomes very small. Yes, if you go directly or face to face, it's different (Participant 2).* Another nursing student also expressed: *There are lecturers who are not very good at using technology, for example we use Moodle right, sometimes their materials can't be opened because they are locked, they haven't opened the date (Participant 4).*

Moreover, the sub category of “remote area” also became a stress source to the nursing student. The network is also burdensome for nursing students as stated by one nursing student: *Poor network often makes it difficult for us to catch the material or we can't go to class because the signal is not good, it makes our understanding go down, maybe because of my place in the village (Participant 9)*. Another nursing student stated: S*ometimes maybe because of my location, which makes when I'm asking our signal broken, so the questions and answers don't connect (Participant 15).*

The category of “Internal factor declined,” contained experiences of nursing student’s individual responses to the new online learning system. Nursing students’ motivation weakened during the online learning period, as it is reflected by one nursing student: *Learning feels very relaxed, don't have to take a shower first, just wear a t-shirt, sometimes gets too lazy, motivation to study decreases (Participant 17)*. 

### Topic of Coping Strategy

#### Seeking Support Theme

This theme contained experiences of seeking support from government, faculty, peers and family. In the category of “government support,” the minister of education supported it as a form of quota support for education. Nursing students explained their coping strategy such as: *Use the learning quota facility from the government effectively. We get quota assistance from the government and it goes on time every month to our cell phones (Participant 11)*. In addition, in the category of “faculty support,” nursing students experience of coping strategies by seeking support from faculty. Their coping strategies included maintaining relationships with the faculty. Though using technology, nursing students are trying to get closer with the faculty, as stated by one nursing student: *Trying to get closer, use effective communication via chat, if there is something I don't understand, I chat with the lecturer and the lecturer immediately answers my chat (Participant 12)*. Another nursing student in the internship stage expressed: *This is for me personally, so for example, zooming in was not clear, while the time is up, I'm going to chat again, chat again, chat again (Participant 14)*. On the other hand, the sub category “enjoying learning,” nursing students were able to use some situations for leisure. Therefore, some students reflected: *The relaxed situation in online learning such as lying back while off camera or eating is not a problem for some lecturers (Participant 1).* Another nursing student at the internship stage stated: *Although online learning is a source of stress, students can use some learning situations as a means of fun, take it easy and relaxed, want to start class, just open the laptop (Participant 11).*

In the category “peer support” nursing students were seeking support from classmates in seeking cooperation and collaboration with friends, as reflected by a nursing student at internship stage: *Inviting friends to discuss cases, its cooperation, right, sharing and discussing the exercises or questions given by the lecture (Participant 10).* Another nursing student expressed: *For example, the lecturer divides groups in doing assignments, or while zooming in with the lecturer, also chatting with friends to discuss what the lecturer means (Participant 1)*. Moreover, the sub category “Joke with each other” was experienced by the nursing students to seek peer support while encountering stress on online learning. Nursing students state that they joke with each other before starting a zoom class or during a zoom class. Some lecturers give students the opportunity to throw jokes at each other in zoom. 

In the category “family support” means that nursing students receive support from family such as parents or brothers and sisters in engaging in online learning. This is considered by nursing students as a means to reduce stress in online learning. One nursing student at academic stage shared: *When studying at home, there are mama and papa, so if I don't know, I can go to them, if you’re offline, you can’t (Participant 2)*. Another nursing student reflected: *Closer to the family, more time to rest, even though there are many tasks, you can relax, right (Participant 9).*

#### Positive Acceptance Theme

This theme is described by the experience of nursing students as an effort to cope with stress due to a drastic change from traditional learning to online learning. Students at the internship stage show more meaningful adaptation in dealing with the stress of online learning. Most of them gave a positive response and behavior in dealing with the online learning environment. In regard to positive responses, some reflected that though online learning, they still gain knowledge. One example statement: *I think there are more positives, because even in this situation, even though we are online, we can still gain knowledge online, right? (Participant 13).* In regard to positive behavior, some revealed that they keep on learning with enthusiasm and gratitude, and take valuable experiences in online learning. One example statement: *For example, we take the positive things, we accept the situation we live in with God's help, everything can be done (Participant 17*).

## Discussion

The findings of this study showed that almost one out of two students had some level of stress (16% between severe and extremely severe) range of stress levels, they were encountered with various forms of stressors. Nursing students in both the academic and internship stages were faced with changes in the learning system from traditional learning to online learning. This agrees with a previous study who found nursing students considered online learning very stressful during the COVID-19 outbreak. Several factors associated were found as devices used, stability of internet connection, income status, and geographic area.^24^ These all contribute to the stress level of nursing students in online learning. 

The academic stage of the bachelor nursing program is not surprising that at this stage it is more towards academic completion. Students are expected to meet learning outcomes that refer to the National Higher Education Standards 2015, for level 6, bachelor degree. The learning outcomes encompasses aspects of general attitudes and skills as contained in the Nurses Education Curriculum.^25^ On the other hand, the internship stage of the bachelor nursing program is a professional nursing education in Indonesia. It is an advanced stage of education from academics in the undergraduate nursing program. The students will experience an adaptation process of professional nursing that is able to accept delegation of authority to carry out nursing care. Based on the Nurses Education Curriculum, the internship program is a clinical practice based. Nursing students expected to be able to apply theories and concepts obtained during the academic stage in the form of practice. However, in the COVID-19 pandemic era, it was replaced with online learning. Undoubtedly, stress perceived by the nursing students.^26^

The three coping strategies utilized by the nursing students in both academic and internship stages in this study were emotion focused, problem focused and used together in a balanced way. While most nursing students utilized emotion focused coping strategies, fewer nursing students utilized both the problem and combination of the two coping strategies. This finding is suitable to previous studies that validated emotion focused and problem focused coping strategies. While emotion focused coping encompasses the processes that serve to reduce emotional distress, problem focused strategies look to change the situation for the better. The emotion focused coping covers the acceptance, positive restructuring, and humor, whilst problem-focused coping covers generating alternative solutions, planning and taking action to resolve or circumvent the stressor.^27^ In the qualitative results, the theme of stress source “new experience” emerged from the two categories “ineffective learning process,” and “ineffective interaction.” The other theme of stress sources, “hindrances to online learning,” emerged from the category “external impediments,” and “internal factor declined.” A prior study found that students’ experiences of the transition from face-to-face to e-learning in the context of COVID-19 pandemic.^28^ Another study showed that nursing students experienced technological challenges, academic relationship changes, role stress/ strain and student resilience.^29^ Another study highlighted the students’ responses about online learning experienced by students in the COVID-19 pandemic era. Several aspects found including the positive and negative impact of online learning, economic conditions and anxiety during online learning.^30^ These studies described earlier supported the recent qualitative findings.

The qualitative finding of coping strategies utilized by nursing students covers the theme “seeking support,” that emerged from several categories including government, faculty, peer and family support. The theme “positive acceptance,” emerged from the category “personal adaptation.” These results are consistent with the findings of previous studies. Seeking information and consultation were found as a possible coping strategy for nursing students while encountering stress in online learning during COVID-19 pandemic era. In addition, this study noted that maintaining a positive attitude in seeking information and consultation was a positive coping strategy associated with better mental outcomes among nursing students.^31^ Another study validated the viable strategy for positive coping is accepting attitudes towards online learning positive coping strategies including active coping, positive re-framing, planning and acceptance.^32^^,^^33^

In conclusion, the findings of this study showed that most nursing students were at the normal range of stress level, yet could not be denied they encountered the stressors while engaging in online learning in COVID-19 pandemic era. Nursing students both in the academic and internship stage preferred to utilize emotion focused coping strategies. The new experience and hindrances to online learning became the source of stress in online learning. While new experience stressors came from ineffective learning process and ineffective interaction during online learning, hindrances to online learning came from external impediments and internal factors declined. The coping strategies such as seeking support and positive acceptance were used to deal with stressors during online learning. The government, faculty, peer and family support gave students strength to survive while encountered with stressors. In addition, personal adaptation was used as a positive coping strategy to deal with stress.

Relevance to clinical practice. This study provides relevant information on what could help improve teaching and learning process in online learning for students and thus have positive impact on the quality of care for patients. While there is a growing body of research on stress and coping mechanisms in online learning, a need to continue to study it through thoughtful, well-designed studies to serve as a guide to scholars and educators in online learning and beyond.
